# Supplementary material for: Ectopic Expression of Gs5PTase8, a Soybean Inositol Polyphosphate 5-Phosphatase, Enhances Salt Tolerance in Plants
Source: Int J Mol Sci. 2020 Feb 4;21(3):1023. doi: 10.3390/ijms21031023 (PMC7037738; doi:10.3390/ijms21031023)
Supplement: Supplementary file 1 [file ijms-21-01023-s001.pdf]

Table S1. Sequences of primers.

Name	Sequences (5' to 3')	Gene	Used for
JO36F1	CCTCTAGACAATGCCGTTGACTCACGTT	*Gs5PTase8* (XM_028369920) / *Gm5PTase8* (*Glyma.03G173000*)	cDNA cloning
JO37R1	GGCTCGAGCCGCGAGCTACGGTTACTT		
JO109F2	CCTCTAGAATGAGAACAGAATCAAAGAAG		Cellular localization
JO110R2	GGCTCGAGAAACCTAGGTATGGTGTCTATG		
JO101F	CCACTATGTTGCCCAATCCT		qRT-PCR
JO102R	ACATCTGATTCAGGCCTTCC		
GmELF1bF	CCACTGCTGAAGAAGATGATGATG	*GmELF1b* (*Glyma.02G276600; Glyma.14G039100)*	qRT-PCR
GmELF1bR	AAGGACAGAAGACTTGCCACTC		
NtL25F1	CCCCTCACCACAGAGTCTGC	L25 ribosomal protein	qRT-PCR
NtL25R1	AAGGGTGTTGTTGTCCTCAATCTT		
Act2F	ACATTGTGCTCAGTGGTGGA	*AtACT2* (*At3g18780*)	qRT-PCR
Act2R	TCATACTCGGCCTTGGAGAT		
NPTIIF	TCCATCATGGCTGATGCAAT	*NPTII* (kanamycin selection marker)	Geno-typing
NPTIIR	CGATACCGTAAAGCACGAGG		
JO105F	CTGGATCCATGGTGAGCAAGGGCGAGG	*GFP*	Cellular localization
JO106R	GGCTCGAGTTACTTGTACAGCTCGTCC		
JO166F	AGTTGACTCCGGTTTACGAA	*AtRD29B* (*At5G52300*)	qRT-PCR
JO167R	TCTCCAGGTTTCAGCTTCTC		
JO168F	GAGCAACGAGGGGAAGATAA	*AtRD29A* (*At5G52310*)	qRT-PCR
JO169R	CTTCCTTTGTCGTCGTTTCC		
JO170F	GCTGGGGTAAAGAAGTTGTC	*AtRD22* (*At5G25610*)	qRT-PCR
JO171R	TTCCAAGCTGAGGTGTTCTT		
JO172F	ACATCCAGGAAGAGTTGGTG	*AtABA1* (*At5G67030*)	qRT-PCR
JO173R	TACGTTCAAGAGCATCGTCA		

F in the name of primers presents for forward primer and R presents for reverse primers.

Figure S1.

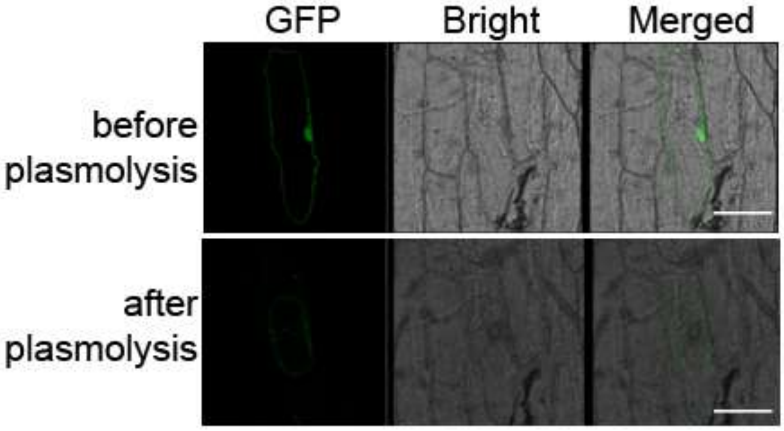

Figure S1. Subcellular localization of Gs5PTase8 in another transformed onion epidermal cells. Gs5PTase8-GFP driven by CaMV 35S were transiently expressed in onion epidermal cells and observed before or after plasmolysis. GFP fluorescence, bright-field and merged images are shown. Scale bars indicate 100 μm.
